# Bile acids increase intestinal marker expression via the FXR/SNAI2/miR-1 axis in the stomach

**DOI:** 10.1007/s13402-021-00622-z

**Published:** 2021-09-12

**Authors:** Na Wang, Siran Wu, Jing Zhao, Min Chen, Jiaoxia Zeng, Guofang Lu, Jiaojiao Wang, Jian Zhang, Junye Liu, Yongquan Shi

**Affiliations:** 1 Xijing Hospital of Digestive Diseases, State Key Laboratory of Cancer Biology, Fourth Military Medical University, No. 15 West Changle Road, Xi’an, 710032 Shaanxi China; 2 Xi’an Jiaotong University, Xi’an, China; 3 Shannxi University of Chinese Medicine, Xi’an, China; 4 Department of Radiation Protective Medicine, Fourth Military Medical University, No. 15 West Changle Road, Xi’an, 710032 Shaanxi China

**Keywords:** Gastric cancer, Intestinal metaplasia, FXR, SNAI2, CDX2

## Abstract

**Purpose:**

Intestinal metaplasia (IM) is a precancerous lesion that increases the risk of subsequent gastric cancer (GC) development. Previously, miR-1 has been shown to play an essential role in the initiation of bile acid (BA)-induced IM. The objective of the present study was to investigate the mechanism underlying miR-1 inhibition by BA in gastric cells.

**Methods:**

Ingenuity pathway analysis (IPA) was used to identify molecules acting upstream of miR-1. The effects of deoxycholic acid (DCA), FXR and SNAI2 on the expression of intestinal markers were assessed using quantitative real-time PCR (qRT-PCR) and Western blotting. The expression level of major molecules was detected by immunohistochemistry (IHC) in tissue microarrays. The transcriptional regulation of miR-1 was verified using luciferase reporter and chromatin immunoprecipitation (ChIP) assays.

**Results:**

We found that BA treatment caused aberrant expression of FXR and intestinal markers in gastric cells. Augmented FXR led to transcriptional activation of SNAI2, which in turn suppressed the miR-1 promoter. Moreover, we found that compared with normal tissues, the expression levels of both FXR and SNAI2 were increased and positively correlated with each other in IM tissues. Additionally, their expression showed an inverse correlation with that of miR-1 in IM tissues.

**Conclusions:**

Our findings indicate that FXR may be responsible for a series of molecular changes in gastric cells after BA treatment, and that the FXR/SNAI2/miR-1 axis exhibits a crucial role in BA-induced progression of IM. Blocking the FXR-oriented axis may provide a promising approach for IM or even GC treatment.

**Supplementary Information:**

The online version contains supplementary material available at 10.1007/s13402-021-00622-z.

## Introduction

Gastric cancer (GC) is the fifth most common malignant tumor and the third leading cause of cancer-related death worldwide [1]. Researchers have gradually recognized that gastric intestinal metaplasia (IM) is one of the most common precancerous lesions of intestinal-type GC, which progresses from chronic atrophic gastritis, IM, and atypical hyperplasia to GC [2, 3]. Preventing the development of IM may block the development of intestinal-type GC. As yet, however, the mechanism underlying the occurrence of IM in the gastric mucosa is still unclear.

It is generally believed that chronic environmental stimulation such as *Helicobacter pylori* (*Hp*) infection and bile acids (BA) reflux leads to gastric IM [4, 5]. However, whether *Hp* eradication can prevent the occurrence or further development of GC is still controversial [6, 7], indicating that other factors may contribute to the development of IM. BAs are important factors inducing or exacerbating IM [8]. Interestingly, many studies have shown that BA stimulation can cause IM in the esophagus, and the underlying mechanism has been largely revealed [9–12]. However, few studies have examined the mechanism underlying the pro-metaplastic role of BAs in gastric cells. According to clinical results, the risk of gastric IM in patients with bile reflux is 11 times higher than that in patients without bile reflux [13]. However, the mechanism by which BAs promote gastric IM requires further study.

MicroRNAs (miRNAs) can cause degradation of mRNAs or inhibition of translation by binding to the 3′ noncoding regions (3′-UTR) of target mRNAs and, thereby, play important roles in regulating biological processes in cells [14, 15]. At present, many studies have provided evidence that miRNAs may participate in the development, differentiation and formation of the digestive tract and, thus, they may also have influence on the occurrence of IM. These miRNAs include miR-30, miR-194 and miR-490 [16, 17]. Moreover, we previously successfully constructed a cell model of BA-induced gastric IM and found that miR-92a promotes the expression of the IM markers Caudal-related homeobox2 (CDX2), Krüppel-like factor 4 (KLF4) and VILLIN 1 (VIL1) by targeting FOXD1 [18]. We also found that the expression of miR-1 was significantly reduced in tissues of patients with IM and in cell models of BA-induced IM, and that the reduction in miR-1 significantly increased CDX2, MUC2 and KLF4 expression by targeting both HDAC6 and HNF4α [19]. However, the mechanism by which BAs trigger miR-1 downregulation remains to be elucidated. In the current study, we performed ingenuity pathway analysis (IPA) between miR-1 and the potential BA receptors Farnesoid X Receptor (FXR, also known as NR1H4), Takeda G-coupled Protein Receptor 5 (TGR5), Small Heterodimer Partner (SHP), Constitutive and Rostane Receptor (CAR) and Vitamin D Receptor (VDR). Our results suggest that FXR may be an interacting protein.

FXR, a BA-activated receptor, is known to be predominantly expressed in the intestine and liver to maintain BA homeostasis [20, 21]. FXR can also serve as a transcription factor to regulate the expression of downstream genes by directly binding to their promoters. With increasing recognition of the important role of BAs in the induction of IM of gastric mucosa, researchers have begun to study the role of FXR in this process. Yu et al. found that upregulation of CDX2 and MUC2 in BA-induced gastric IM cells results from activation of the FXR/NF-κB pathway [22]. Similarly, we previously found that FXR is responsible for enhancement of CDX2 expression in a SHP-dependent manner in gastric cells [23]. However, the mechanisms by which FXR promotes BA-induced gastric IM have not been fully elucidated.

Here, we show that the FXR/SNAI2/miR-1 axis is markedly activated in gastric cells after BA treatment and triggers an intestinal-like phenotype. Based on our results and recent findings suggesting a significant role of FXR in IM and tumor development, we hypothesize that FXR may be responsible for molecular changes resulting from BA stimulation and may, subsequently, facilitate IM to GC progression.

## Materials and methods

### Cells and culture conditions

The BA-induced cell model of IM has been described previously [18]. GES-1 and AGS cells (purchased from ATCC) were cultured in RPMI-1640 medium (#11875093, Gibco, USA) supplemented with 10% fetal bovine serum (#10099141, Gibco, USA), streptomycin and penicillin (#0110AP-100, InCellGene, USA). Primary cells were incubated in an ICell Primary Epithelial Cell Culture System (#icell-001, iCellBioscience, China) with 2% fetal bovine serum (#IC-1905, BiocytoSci, China). Deoxycholic acid (DCA, incubating time: 24 h, dosage: 100 μM) was chosen for the different experiments because it is a major hydrophobic bile acid with potent cytotoxicity (#IC-015563, BiocytoSci, China).

### Tissue microarray and human IM samples

A normal tissue microarray containing samples from 24 subjects (BN01011b) and a gastric IM tissue microarray (ST8017a) containing samples from 80 gastric IM cases were purchased from Alenabio Biotech (China). Ten pairs of biopsies from gastric mucosa were obtained from patients who underwent gastroscopy. The pathological data of these latter specimens were collected from the Department of Pathology. Our study was approved by the ethics committee of Xijing Hospital. All patients signed informed consent forms before the specimens were obtained. To exclude the effect of *Hp* infection, we selected patients who were *Hp* negative.

### Immunohistochemistry (IHC) assay

Tissue slides were incubated at 60 °C for 4 h before deparaffinization and rehydration. The slides were boiled for 2 min in a pressure cooker filled with sodium citrate to retrieve antigen. Anti-FXR (#187735, 1:50, Abcam, UK) and anti-SNAI2 (#PA5–73015, 1:50, Invitrogen, USA) antibodies were dripped onto the slides and incubated overnight at 4 °C. Subsequently, slides were incubated with secondary antibodies for 40 min at room temperature. Finally, the sections were stained with DAB and hematoxylin, dehydrated and transparentized. The results obtained were scored by two professional pathologists independently. The scoring was the product of the positive rate and intensity of staining. The staining intensity was scored as follows: negative (0), weak (1), moderate (2), and strong (3). The positive rate of staining was scored as <10% (0), 10–25% (1), 26–50% (2), 51–75% (3), and >75% (4).

### Immunofluorescence assay

Cells were seeded in 4-well chamber slides (#PEZGS0416, Millipore, USA), washed twice with cold PBS, fixed with 500 μl 4% paraformaldehyde for 35 min, permeabilized with 200 μl 1% Triton X-100 for 10 min, and blocked with 500 μl goat serum for 30 min. Next, the cells were incubated with primary antibodies at 4 °C overnight. Subsequently, the cells were incubated with FITC-conjugated goat anti-mouse IgG antibody (#12–506, 1:200, Millipore, USA) at room temperature for 2 h. Finally, the nuclei were stained with DAPI (#C0060, 1:800, Solarbio, China) for 10 min. The primary antibody used was a rabbit anti-human FXR antibody (#187735, 1:100, Abcam, UK).

### RNA extraction and qRT-PCR

TRIzol® reagent (#15596026, Invitrogen, USA) was used to extract total RNA from cell lines and primary human tissue samples according to a standard protocol. Then, RNA was reverse-transcribed into cDNA using a PrimeScript® RT Reagent kit or a Mir-X miRNA First-Strand Synthesis Kit (#RR036A, #638315, TaKaRa Biotechnology, Japan) at 37 °C for 15 min and 85 °C for 5 s, followed by temperature maintenance at 4 °C. qRT-PCR was conducted using a SYBR Premix Ex Taq II or a Mir-X miRNA qRT-PCR TB Green Kit (#RR820A, #638314, Takara Biotechnology, Japan) on a CFX96™ Real-Time PCR Detection system (Bio-Rad Laboratories, USA). β-actin and U6 were used as internal controls, respectively, and the 2^∆∆Cq^ method was used to quantify the relative mRNA expression level of each gene. The sequences of the gene-specific primers used are shown in Table 1.

**Table 1:** Sequences of primers used in the study

Primer name	Forward sequence	Reverse sequence
SNAI2	CGGAAGCCTAACTACAGCGA	GGACAGAGTCCCAGATGAGC
miR-1	TAGAAGCTTGCCTCTGAGCTGCCTTCTCTA	
SNAI2 promoter 1	GCGGAAGCCCTGAGTAGCGC	CCTCTGGTGTTAATGAGAGCC
SNAI2 promoter 2	TGTAAAGAGATATATGAGGG	ATGTTATACTTTCTCCAGTACAC
SNAI2 promoter NC	GATGCCCAGGTAAGCCCCCAG	CAAACTTTCAAAGCCAACAC
miR-1 promoter 1	AAGCCTCCTGTGTCTGTGCCCG	TGGAGAAAGAGCCAGAAGGAG
miR-1 promoter 2	AGCTGGTGAGCACCGGGAAATTC	GAAGCGGCAGCTGCGCAGAGC
miR-1 promoter 3	GATGCCCAGGTAAGCCCCCAG	CAAACTTTCAAAGCCAACAC
miR-1 promoter NC	TGAGTGCAGCATAGAGCCCCTG	CAGACGTGGCCATAAATCACC

### Protein extraction and Western blot analysis

Total protein was extracted by mixing cells with radioimmunoprecipitation assay (RIPA) buffer (#P0013B, Beyotime, China), which was supplemented with protease and phosphatase inhibitors. Next, the proteins were separated by SDS-PAGE and transferred to NC membranes (#66485, Pall Inc., USA). The resulting membranes were incubated with 5% milk/TBST for 2 h at room temperature. Next, TBST was used to wash the membranes for 3 × 5 min, after which they were incubated with primary antibodies at 4 °C overnight. Finally, the membranes were incubated with HRP-conjugated secondary antibodies (#SAG10002, AntiProtech Inc., USA) after washing in TBST for 3 × 7 min. The following antibodies were used: anti-FXR (#187735, 1:200, Abcam, UK), anti-SNAI2 (#106077, 1:1000, Abcam, UK), anti-HDAC6 (#7558, 1:1000, Cell Signaling Technology, USA), anti-HNF4α (#92378, 1:2000, Abcam, UK), anti-CDX2 (#12306, 1:1000, Cell Signaling Technology, USA), and anti-β-actin (#AP0060, 1:5000, Bioworld, China). The primary antibody dilution buffer, PREstain Protein Ladder and Western SuperSensitive Substrate were purchased from BioCytoSci (#IC-8008, #IC-8001, China).

### Ingenuity pathway analysis

The gene ID and FC values of the potential BA receptors FXR, TGR5, SHP, CAR and VDR were used as input into the Ingenuity Pathway Analysis (IPA) software (#47547484), which then analyzed the up-down relationships between genes based on literature data and, finally, draws regulatory networks among genes according to their relationships. The references and databases for each gene in the analysis results are shown in Supplementary Information 2, and the list of genes involved in the analysis is shown in Supplementary Information 3.

### Transfection and infection assays

Synthetic miR-1 agomir, the corresponding negative control oligonucleotides and plasmid small interfering RNA (siRNA) were purchased from GenePharma (China), and their sequences are shown in Table 2. Attractene transfection reagent was added to Opti-MEM to generate the transfection medium. Subsequently, cells were cultured in the transfection medium, which was replaced with fresh medium after 6 h. Then, the cells were grown in 5% CO_2_ at 37 °C for an additional 48 h. The transfection reagent was purchased from Thermo Fisher Scientific (USA) and was used following the manufacturer’s protocol. SNAI2 and FXR overexpression lentiviral vectors were designed and provided by GeneChem Co. Ltd. (China). GES-1 cells were infected with the lentivirus for 48 h and then cultured with 2 μg/ml puromycin (#IC-1608, InCellGene, USA) for 7 days.

**Table 2:** Interference targets of each gene involved in the study

siRNA target name	Forward sequence	Reverse sequence
FXR	GGAAGAAAGAAUUCGAAAUTT	AUUUCGAAUUCUUUCUUCCTT
SNAI2	GACCCACACAUUACCUUGUTT	ACAAGGUAAUGUGUGGGUCTT
miR-1 agomir	UGGAAUGUAAAGAAGUAUGUAU	ACAUACUUCUUUACAUUCCAUU

### Dual-luciferase reporter assay

A specific sequence upstream of the transcription start site of the SNAI2 promoter was amplified by PCR and then cloned it into a pGL3-basic luciferase vector (Invitrogen, USA) to generate SNAI2-p1 (−2000 bp ~ +500 bp), SNAI2-p2 (−543 bp ~ +500 bp), SNAI2-p3 (+398 bp ~ +500 bp) and SNAI2-p4 (+415 bp ~ +500 bp). We also amplified the 2000-bp sequence upstream of the transcription start sites of the miR-1 promoter, and then cloned it into a pGL3-basic luciferase vector.

### Chromatin immunoprecipitation assay

GES-1 cells were transiently transfected with a miR-1 promoter vector. Then, ChIP analysis was performed according to the standard method of the Magna ChIP G Assay kit (#16–662, Millipore, USA). Chromatin was immunoprecipitated with anti-FXR (#SC-25309, Santa Cruz, USA), anti-SNAI2 (#9585, CST, USA) or a negative control IgG. Finally, immunoprecipitated DNA-protein complexes were isolated and a real-time PCR assay was carried out. The primers for the SNAI2 and miR-1 promoters are listed in Table 1.

### Statistical analysis

The SPSS software (V.19.0, SPSS, USA) statistical package was used to conduct all statistical analyses. All continuous data are expressed as the mean ± SD. The χ^2^ test was used to compare frequencies of categorical variables. Mutual associations among clinical results were assessed using Spearman’s rank correlation. Statistical comparisons between two groups were analyzed with the unpaired Student’s t test. *P* values < 0.05 were considered statistically significant.

## Results

### FXR expression is increased in intestinal metaplastic cells

**Fig. 1:**
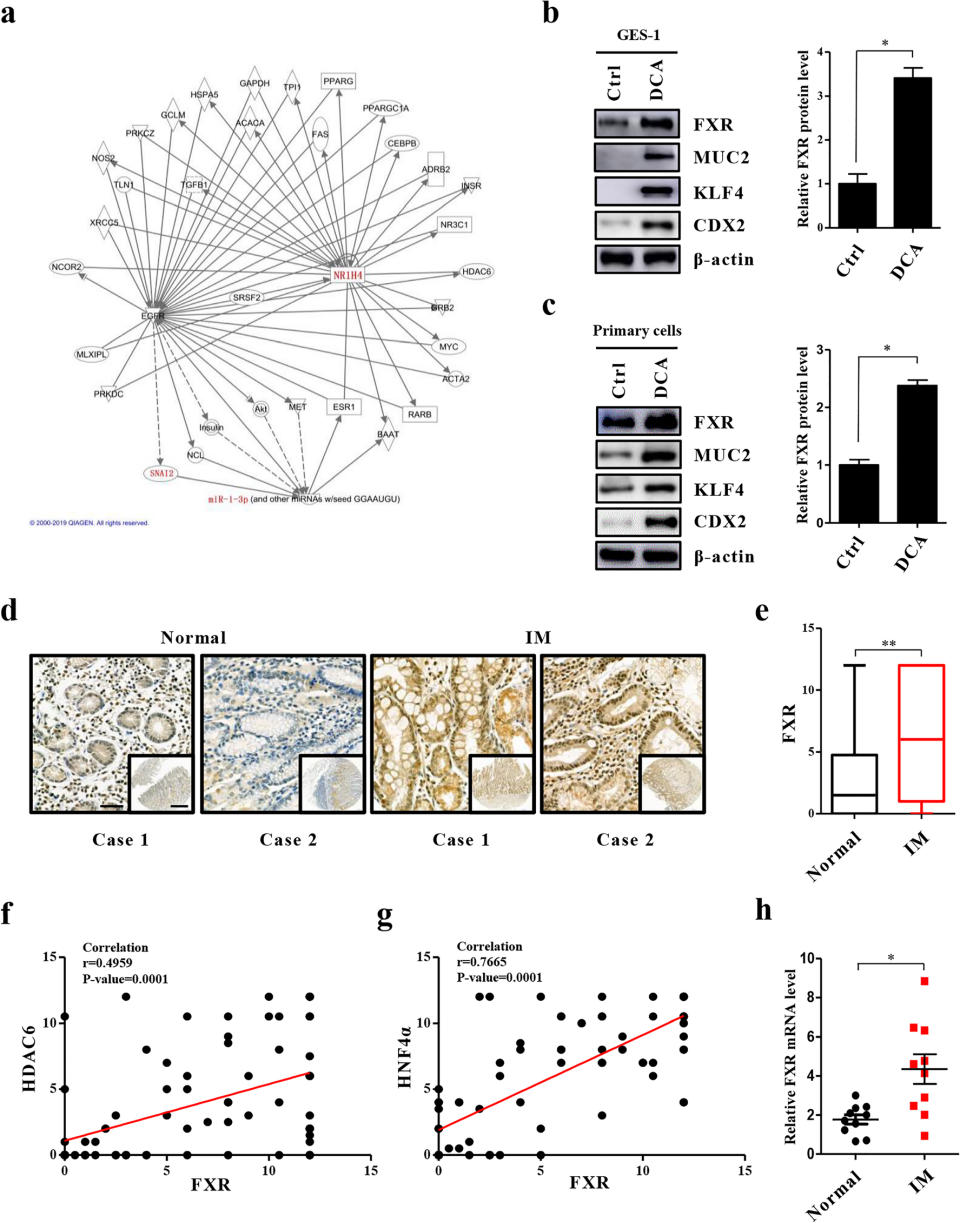
Upregulation of FXR in bile acid (BA)-stimulated intestinal metaplasia (IM) cells and tissues. **(a)** IPA analysis of bile acid receptors related to miR-1. **(b)** Left: Effects of DCA on the expression of FXR and intestinal markers (CDX2, MUC2, KLF4) in GES-1 cells. Cells were stimulated with DCA (100 μM) or vehicle alone for 24 h after which proteins were extracted and subjected to Western blot analysis. β-actin levels were used as internal control. Right: Quantification of Western blot analysis results normalized to β-actin. (**c)** Left: DCA (100 μM) enhances FXR protein levels and intestinal markers in primary gastric epithelial cells. Right: Quantification of Western blot analysis results normalized as in 1b. (**d, e)** Immunohistochemical (IHC) staining for FXR in normal (*n* = 24) and IM (*n* = 80) tissues on microarrays. Scale bars: 100 μm and 500 μm (insets). **(f, g)** Correlation between FXR and HDAC6 or HNF4α expression in IM tissues detected by IHC. X-axis: FXR expression level. Y-axis: HDAC6 expression level (left) and HNF4α expression level (right). **(h)** FXR mRNA levels in 10 pairs of IM specimens and matched normal specimens. Each symbol represents the mean value of an individual patient. Means ± SEM of representative experiments (*n* = 3) performed in triplicate are shown. *p < 0.05; **p < 0.01

Previously, we found that decreased miR-1 expression promotes the trans-differentiation of normal gastric cells into metaplastic cells after BA exposure by targeting both HDAC6 and HNF4α. To uncover the mechanism by which BA inhibits miR-1, we here conducted IPA and found that FXR links BAs to miR-1 (Fig. 1a). Next, we examined the protein levels of FXR in GES-1 cells treated with DCA (100 μM) for 24 h. As expected, DCA caused an increase in FXR expression along with a high expression of CDX2, KLF4 and MUC2 (Fig. 1b). Since we could not achieve the experimental conditions to construct a bile reflux mouse model, we utilized Sprague-Dawley (SD) rat primary gastric epithelial cells treated with DCA to mimic the condition. Similarly, we found that the expression levels of FXR and intestinal markers were enhanced in the primary cultured cells after DCA (Fig. 1c). In addition, immunofluorescence (IF) staining for FXR was performed in GES-1 cells and the results obtained reconfirmed that DCA leads to enhanced FXR expression in gastric cells (SI1 1a). These results indicate that FXR may participate in downstream gene activation induced by BAs.

To additionally determine the significance of FXR in primary IM tissues, we evaluated and analyzed its expression level in normal and metaplastic tissues. A high FXR expression was observed more frequently in the metaplastic tissues and it was mainly located in the nucleus (Fig. 1d, e, Table 3). Moreover, correlation analyses showed that the expression of FXR was negatively related to miR-1 (r = −0.2390, *p* = 0.0328) and positively correlated with the miR-1 target molecules HDAC6 (r = 0.4959, *p* = 0.0001) and HNF4α (r = 0.7665, *p* = 0.0001) (SI1 1b, Fig. 1f, g) in metaplastic tissues. Additionally, we found that the FXR mRNA level was higher in metaplastic cells than in normal cells (Fig. 1h). Western blot results also showed that the expression level of FXR in IM tissue cells was significantly increased, and that its expression level in AGS cells was obviously higher than that in GES-1 cells (SI1 1c). Collectively, these data show that FXR is expressed in normal tissues and that its expression increased in IM. Therefore, these findings suggest that FXR may be involved in the occurrence and development of IM.

**Table 3:** FXR expression in normal and IM tissues

Histological	N		FXR	Score (n)		*P*
		–	+	++	+++	
Normal	24	7	11	4	2	0.0209
IM	80	18	17	16	29	

### FXR regulates the expression of downstream IM markers and miR-1

**Fig. 2:**
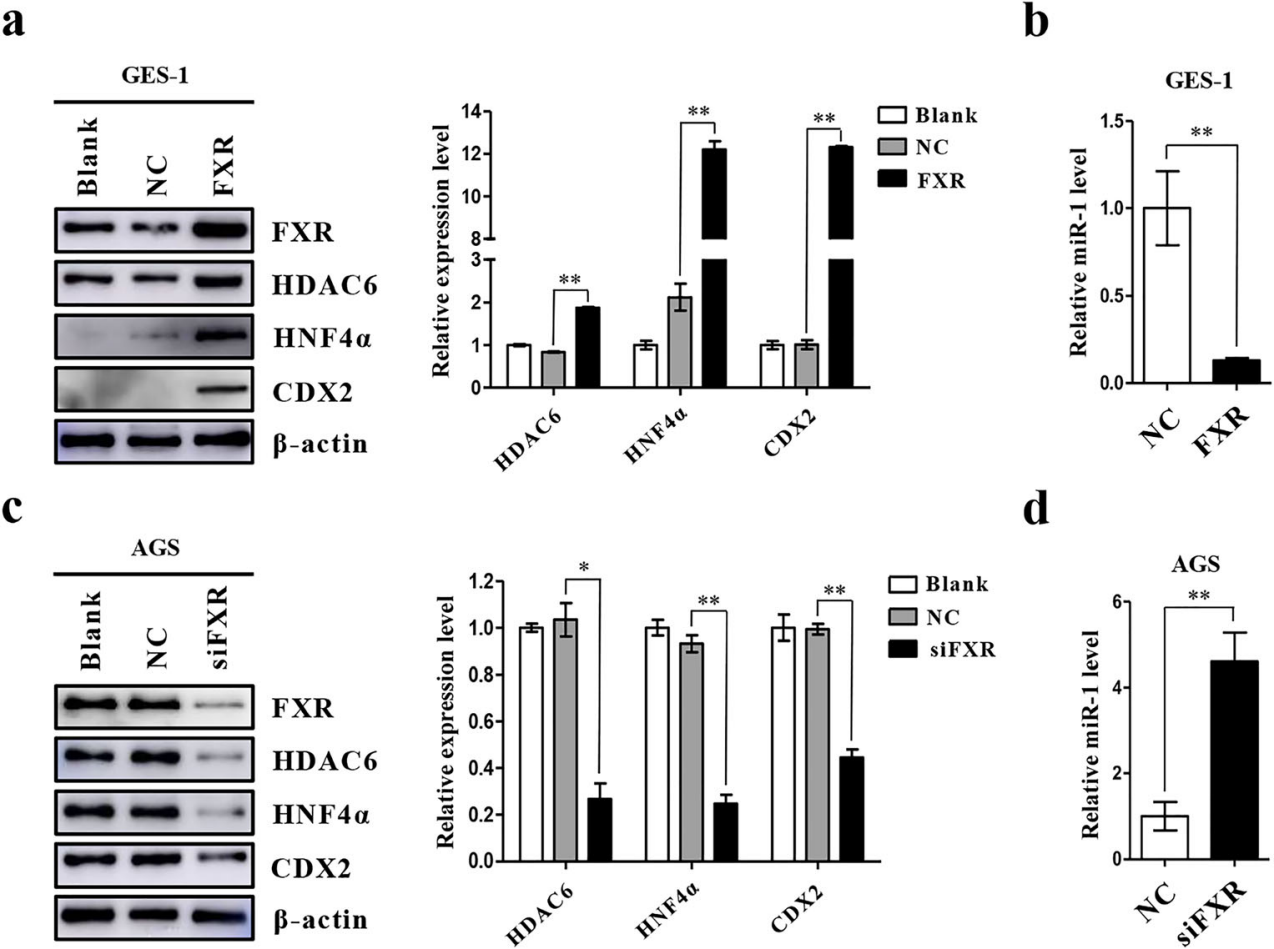
FXR positively regulates expression of intestinal markers in gastric cells. **(a)** Left: Western blot for intestinal markers and miR-1 targets in GES-1 cells infected with a FXR expression vector. Right: Quantification of Western blot analysis results normalized to β-actin. (**b)** miR-1 expression in GES-1 cells detected by qRT-PCR at 48 h post-transfection. U6 was used as an internal control. **(c)** Left: AGS cells were transfected with siFXR and intestinal markers were examined by Western blotting. Right: quantification of Western blot analysis results normalized as in 2a. **(d)** qRT-PCR analysis of miR-1 expression in AGS cells transfected with a FXR-specific siRNA. *p < 0.05; **p < 0.01

We used loss-of-function and gain-of-function experiments to study the role of FXR in regulating intestinal markers. We found that overexpression of FXR in GES-1 cells resulted in an increase in HDAC6 and HNF4α expression, as well as upregulation of CDX2 expression (Fig. 2a, SI1 2a). Moreover, we found that the enhancement of FXR led to downregulation of miR-1 (Fig. 2b). In contrast, we found that the expression of HDAC6, HNF4α and CDX2 decreased significantly after FXR knockdown in AGS cells (Fig. 2c, SI1 2b). The knockdown also caused an augmentation of miR-1 expression (Fig. 2d). Together, these results indicate that FXR may not only positively affect the expression of downstream intestinal markers and miR-1 target molecules, but also suppresses the expression of miR-1.

### SNAI2 is enhanced in BA-induced metaplastic cells

**Fig. 3:**
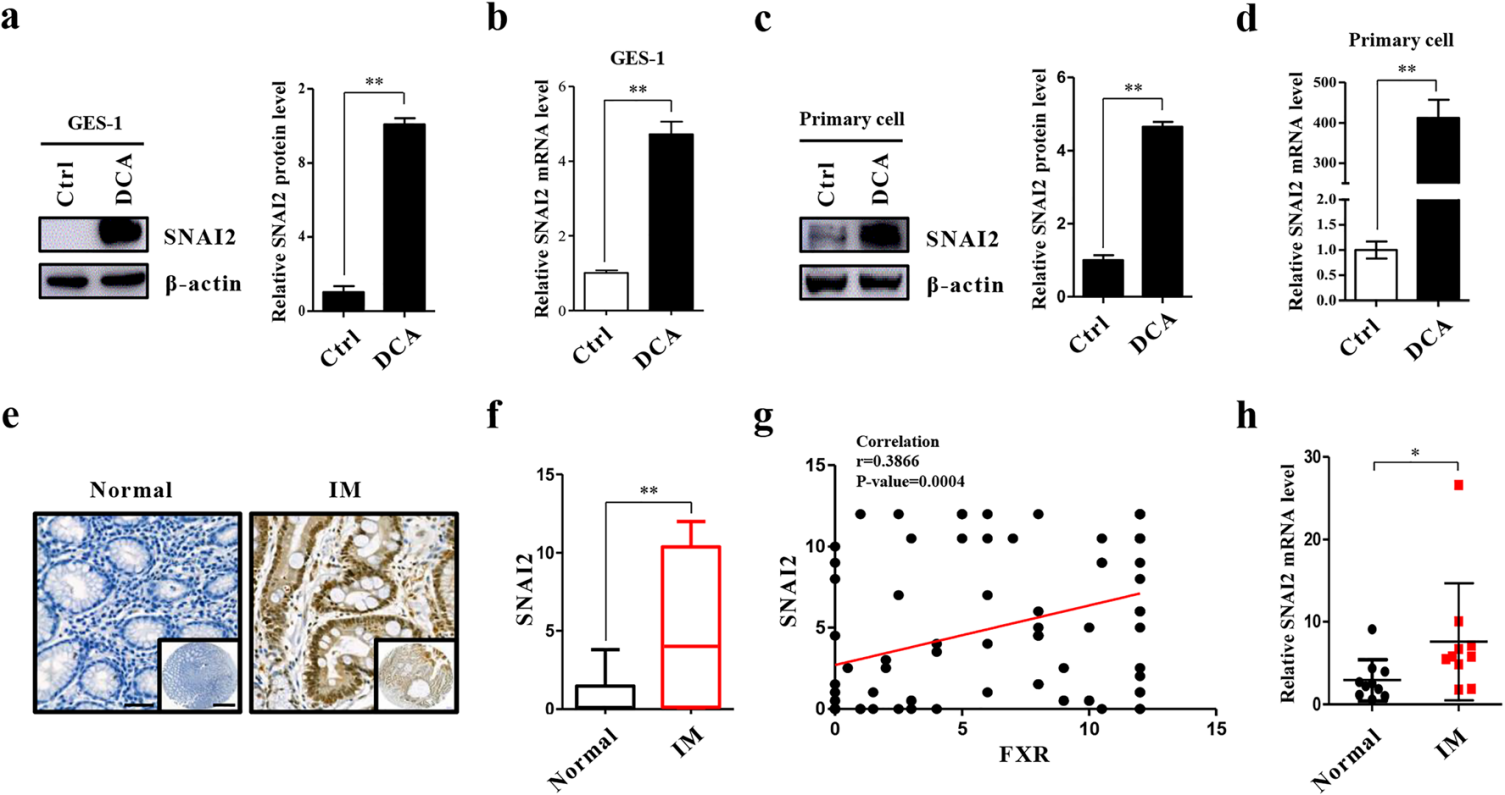
Overexpression of SNAI2 in IM cells. **(a)** GES-1 cells were treated as in Fig. 1b. The protein level of SNAI2 was detected by Western blotting (Left) and the results were normalized to β-actin (Right). **(b)** SNAI2 mRNA levels examined in GES-1 cells after DCA treatment by qRT-PCR. **(c, d)** Primary cultured cells were treated with DCA as for GES-1 cells, after which SNAI2 was detected at protein and mRNA levels, respectively. **(e)** Representative pictures of IHC staining for SNAI2 in normal and gastric IM tissues. Scale bars: 100 μm and 500 μm (insets). **(f)** Quantification of IHC staining for SNAI2. **(g)** Correlation between SNAI2 and FXR expression in IM specimens. **(h)** SNAI2 mRNA levels in 10 pairs of matched human IM/normal specimens. *p < 0.05; **p < 0.01

The results of IPA revealed that FXR may affect the expression of miR-1 through SNAI2, NCL, AKT, and MET. Through bioinformatics analysis, we found that SNAI2, a transcription factor, has binding sites in the promoter region of miR-1. We then set out to detect the protein and mRNA levels of SNAI2 in DCA-treated cells, and found a substantial increase compared with that in control cells (Fig. 3a, b). In addition, we found that SNAI2 was strongly upregulated at both the protein and mRNA levels in primary cells in response to DCA treatment (Fig. 3c, d). We also found that the expression of SNAI2 was enhanced and positively correlated with FXR expression in metaplastic tissues (Fig. 3e–g, Table 4). Its mRNA level was also significantly increased in tissues of patients with IM compared with adjacent normal tissues (Fig. 3h). Taken together, these results indicate that SNAI2 is overexpressed in BA-induced metaplastic cells.

**Table 4:** SNAI2 expression in normal and IM tissues

Histological	N		SNAI2	Score (n)		*P*
		–	+	++	+++	
Normal	24	12	12	0	0	0.0194
IM	80	20	21	14	25	

### SNAI2 is transcriptionally activated by FXR in gastric cells

**Fig. 4:**
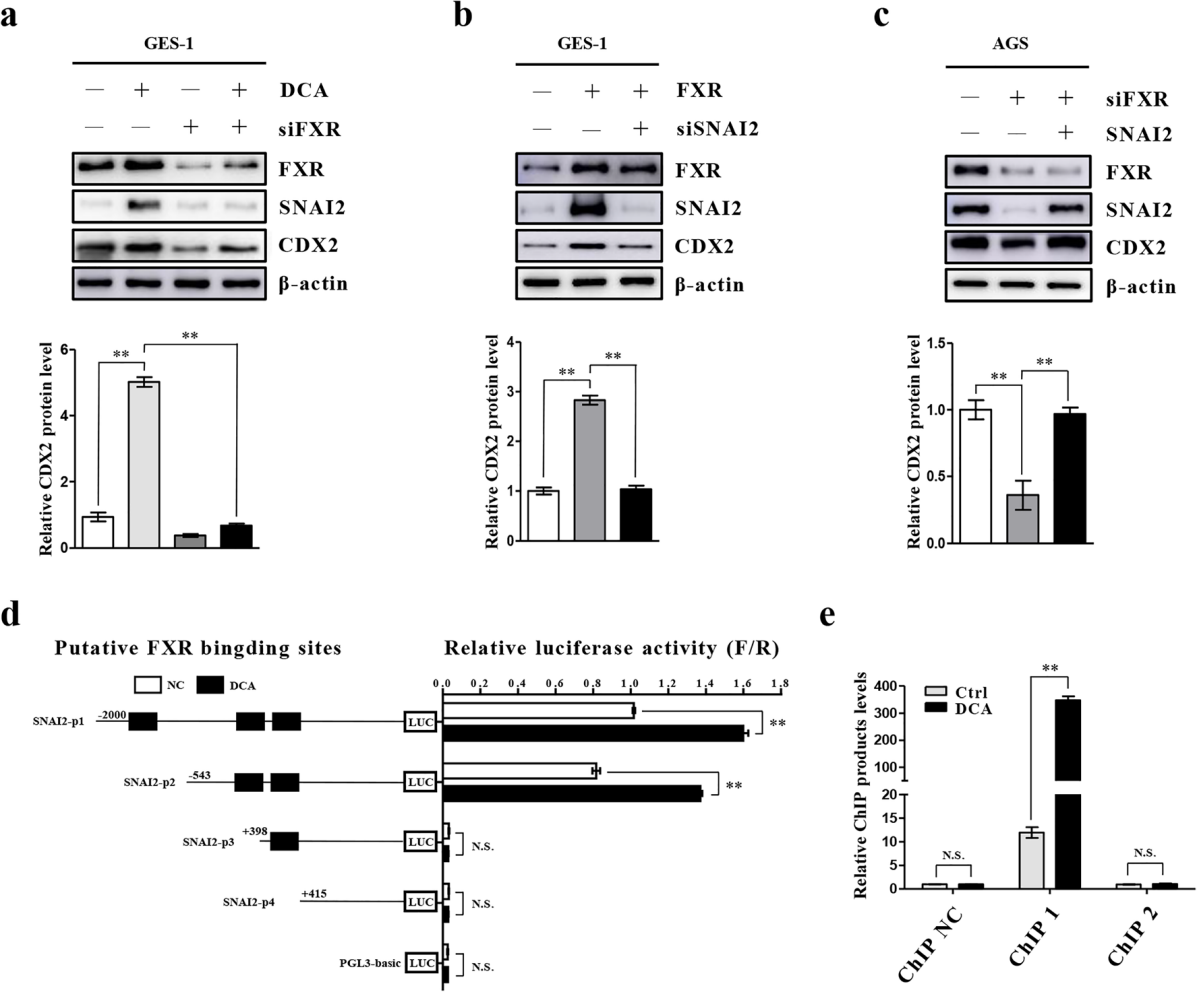
FXR positively regulates SNAI2 transcription. **(a)** Top: FXR inhibition reverses DCA-induced upregulation of SNAI2 and CDX2 in GES-1 cells. Bottom: Quantification of Western blot results normalized to β-actin. **(b)** GES-1 cells were transfected with FXR expression vector and siSNAI2 simultaneously. Top: Downregulation of SNAI2 blocks CDX2 enhancement by FXR overexpression. Bottom: Quantification of Western blot results. **(c)** AGS cells were transfected with SNAI2 expression vector and siFXR. Top: Upregulation of SNAI2 reverses the inhibition of CDX2 by FXR-specific siRNA. Bottom: Quantification of Western blot analysis results. **(d)** Serially truncated SNAI2 promoter constructs were cloned into pGL3-luciferase reporter plasmids and transfected into GES-1 cells. Four hours after transfection, the cells were treated with DCA for 24 h after which relative luciferase activities were determined. **(e)** qRT-PCR analysis of ChIP products validating binding of FXR to the SNAI2 promoter. **p < 0.01. N.S., not significant

To further clarify the role of FXR and SNAI2 in the process of BA-induced IM, we explored the putative regulatory relationship between FXR and SNAI2. We found that CDX2 enhancement induced by DCA could be reversed by a FXR-specific siRNA (Fig. 4a). Next, GES-1 cells were infected with a FXR expression vector and transfected with siSNAI2 simultaneously. We found that the upregulation of SNAI2 and CDX2 induced by FXR could be reversed by siSNAI2 (Fig. 4b). In contrast, we found that FXR silencing resulted in a decreased SNAI2 and CDX2 expression in AGS cells, whereas no decrease was observed after overexpression of SNAI2 (Fig. 4c). According to the JASPAR database prediction results (http://jaspar.binf.ku.dk/), we constructed a series of reporter gene vectors containing different sequences upstream of the SNAI2 transcription start site. We found that the activities of SNAI2-p1 and SNAI2-p2 were higher than those of the other sequences without any treatment, and that their transcriptional activities were significantly increased in GES-1 cells after DCA treatment (Fig. 4d). Subsequent ChIP assays indicated that the ChIP 1 (GAGGTAATTAT) sequence may be the putative binding site of FXR in the SNAI2 promoter (Fig. 4e, SI1 3a). The above results indicate that FXR can promote the transcription of SNAI2 in BA-induced metaplastic cells.

### SNAI2 promotes IM by transcriptionally inhibiting miR-1

**Fig. 5:**
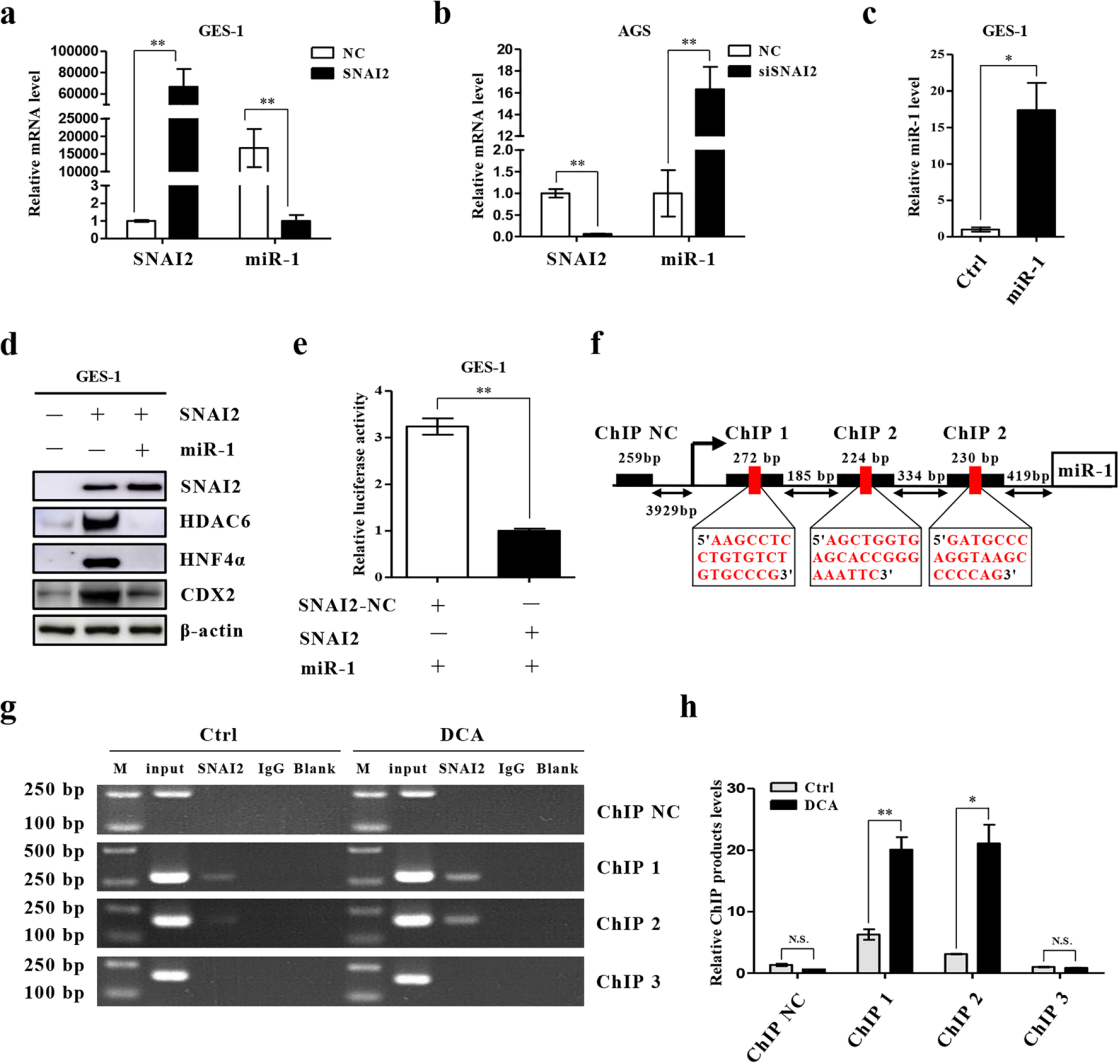
SNAI2 suppresses miR-1 transcription in BA-induced IM cells. (**a, b)** qRT-PCR analysis of SNAI2 and miR-1 expression in GES-1 and AGS cells with SNAI2 overexpression or knockdown. **(c)** miR-1 expression enhancement by ago-miR-1 (100 nM) in GES-1 cells. (**d)** Upregulation of intestinal markers and miR-1 targets induced by SNAI2 in GES-1 cells is abolished by miR-1 overexpression. **(e)** SNAI2 inhibits miR-1 promoter activity in GES-1 cells. **(f, g)** ChIP assay showing direct binding of SNAI2 to the miR-1 promoter in GES-1 cells. M: Marker. **(h)** qRT-PCR of ChIP products validating binding of SNAI2 to the miR-1 promoter. *p < 0.05; **p < 0.01. N.S., not significant

To further uncover the mechanism underlying the pro-metaplastic function of SNAI2, we examined its putative regulation of downstream intestinal markers and miR-1. Initially, we detected the expression level of miR-1 in GES-1 and AGS cells and found that the expression level in GES-1 cells was significantly higher than that in AGS cells (SI1 3b). We also found that high SNAI2 expression resulted in decreased miR-1 expression in GES-1 cells (Fig. 5a). Conversely, we found that miR-1 was augmented after downregulating SNAI2 (Fig. 5b). When SNAI2 and miR-1 were simultaneously overexpressed, miR-1 reversed the increase in HDAC6 and HNF4α expression induced by SNAI2 (Fig. 5c, d). Furthermore, to ascertain whether SNAI2 can regulate the activity of the miR-1 promoter, we co-transfected a SNAI2 expression vector with a miR-1 full-length promoter reporter gene in GES-1 cells. We found that SNAI2 could markedly reduce miR-1 promoter activity (Fig. 5e). Next, we constructed different promoter sequences: ChIP 1 (−1660 bp ~ −1389 bp), ChIP 2 (−1205 bp ~ −982 bp), ChIP 3 (−649 bp ~ −420 bp) and ChIP NC (−5930 bp ~ −5672 bp). ChIP assays were performed to detect the binding sites of SNAI2 to these sequences. We found that the sequences of both ChIP 1 and ChIP 2 could bind to the SNAI2 promoter directly, and that their combination was reinforced by DCA (Fig. 5f–h). In summary, our findings suggest that SNAI2 overexpression in BA-induced metaplastic cells contributes to a reduction in miR-1 promoter activity and an increase in intestinal marker expression.

## Discussion

Through our study we revealed aberrant expression of FXR and SNAI2 in intestinal metaplastic cells and their key roles in BA-stimulated metaplastic progression. Interestingly, we found that SNAI2 negatively regulated the transcription of miR-1, which further targeted downstream intestinal markers. We conclude that the FXR/SNAI2/miR-1 axis mediates the expression of intestinal markers in metaplastic cells. In addition, we found that this axis, when triggered by DCA may promote trans-differentiation of normal gastric cells and mediate further development of IM to GC.

The mechanism of intestinal differentiation of gastric cells is at present not clear. It is widely accepted that IM results from interactions of several intestine-related transcription factors, and that CDX2 plays an indispensable role in stimulating intestinal differentiation [24–28]. Recently, the regulatory role of CDX2 in intestinal differentiation has attracted attention. In a previous study, we found that increased miR-92a expression is involved in CDX2 upregulation and downstream intestinal markers in gastric cells treated with BAs [18]. In addition, our in vivo and in vitro experiments provided evidence that miR-1 downregulation may facilitate the formation of a positive feedback loop involving HDAC6/HNF4α and next stimulate CDX2 expression in BA-induced metaplastic cells. Here, we sought to elucidate the regulatory mechanism of DCA on miR-1 and to establish a molecular network of alterations during BA-induced progression of gastric IM. GES-1, an immortalized gastric cell line, is regarded as a nonmalignant cell line that mimics gastric epithelial cells. In addition, we identified changes in target molecules in gastric mucosa cells of SD rats treated with BAs.

A retrospective study conducted in Japan found that the incidence of GC in patients with high BA levels in gastric juice (≥1000 mmol/L) was significantly higher than that in patients with low gastric BA levels (<1000 mmol/L) [13]. Another multicenter study showed that the risk of GC was 2.4 times higher in the high concentration BA level group than in the other groups [8]. FXR regulates the biosynthesis, secretion and transport of BAs and plays a role in various metabolic diseases [29]. This molecule not only acts as the principal nuclear receptor of BAs, but also a transcription factor that regulates gene expression [30]. Therefore, we speculated that it might be involved in the stimulation of gastric cells by BAs. In this study, we found that the expression level of FXR increased in intestinal metaplastic tissues as well as in GES-1 cells and primary cultured cells after BA treatment. We also provide evidence that FXR may stimulate the expression of downstream intestinal markers and that FXR silencing leads to abolishment of the enhancement of these markers induced by DCA. Concordantly, other studies have shown that FXR is augmented in intestinal-type GC with IM and contributes to an increase in MUC2 and CDX2 expression in gastric cells [31, 32]. Hence, we hypothesize that FXR may mediate the pro-metaplastic effect of DCA on gastric cells.

Based on IPA and bioinformatics analysis, SNAI2 was considered to link FXR with miR-1. SNAI2 (also known as Slug), a member of the Snail superfamily, is one of the transcription factors involved in epithelial mesenchymal transition (EMT) [33]. In recent years, SNAI2 has been shown to be abnormally expressed in various malignant tumors and to play an important role in tumor progression [34–36]. To investigate the role of SNAI2 in intestinal metaplastic cells, we assessed its expression in metaplastic tissues and BA-induced cells. The results confirmed that it was positively correlated with FXR in metaplastic tissues and could be activated by DCA in GES-1 cells as well as in primary cultured gastric cells. We also analyzed the sequence of the SNAI2 promoter and found that FXR might have binding sites in the promoter. Subsequent ChIP and luciferase reporter assays confirmed that FXR could directly bind to the SNAI2 promoter and activate its transcription. Increased IM marker expression via FXR overexpression or DCA treatment could be blocked by SNAI2 knockdown, indicating that it can mediate the pro-metaplastic function of DCA. We also provided evidence that SNAI2 can stimulate the expression of intestinal markers in gastric cell lines and primary cultured gastric cells. These results are at least partly consistent with some other studies showing that SNAI2 promotes the malignant transformation of gastric cells in an EMT-dependent manner [37–40]. In this study, we showed that SNAI2 is augmented in IM tissues and can promote the expression of intestinal markers in gastric cells.

In terms of the downstream marker regulation, miR-1 was identified as a functional target of SNAI2 by bioinformatics analysis. Dysregulation of miR-1 was found to contribute to the progression and poor prognosis of human gastric cancer [41]. More interestingly, miR-1 was previously found to post-transcriptionally regulate SNAI2 to inhibit EMT in different cancer types [42, 43]. Here, we showed that SNAI2 directly binds to two sites (−1205 bp ~ −982 bp and − 649 bp ~ −420 bp) within the miR-1 promoter to inhibit its transcription. Previously, we showed that miR-1 could target the 3′-UTR of both HDAC6 and HNF4α to suppress their expression. Moreover, DCA inhibited miR-1 in gastric cells to induce high expression of HDAC6 and HNF4α, which stimulated intestinal marker expression. Together with the results reported herein, we identified a complex signaling network through which DCA induces the occurrence and continuous progression of IM in the stomach. Collectively, these data provide evidence that BA-induced gastric IM is achieved, in part, via the FXR/SNAI2/miR-1 axis. The discovery of this pathway may help us to further understand the mechanism of IM, and suggests that we may eventually prevent the occurrence of IM or its further development into GC by blocking FXR activation. Notably, model systems of bile reflux, such as mouse models or primary cells from human gastric mucosa, will be needed in future studies.

**Fig. 6:**
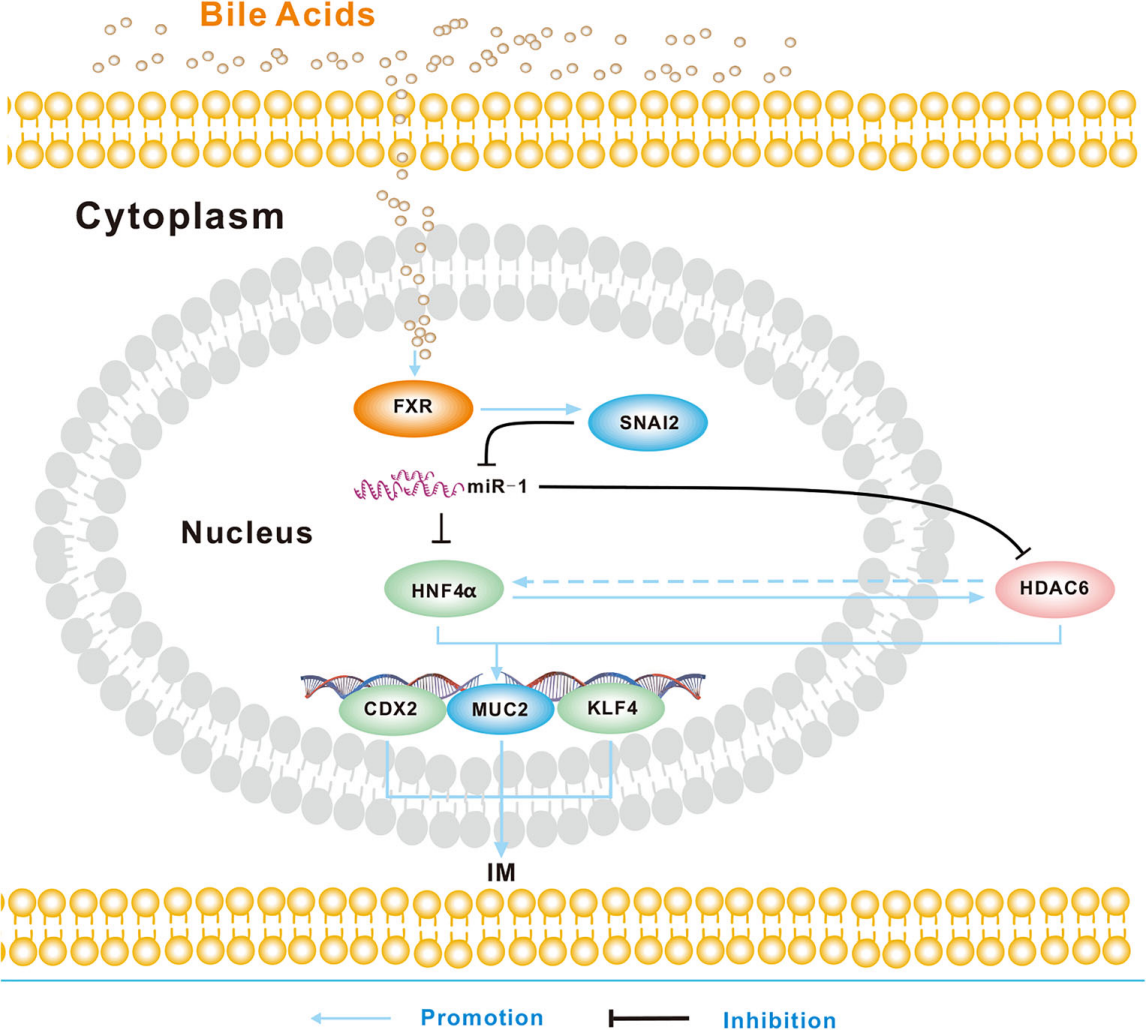
FXR/SNAI2/miR-1 signaling pathway promotes BA-induced gastric IM. Under the stimulation of bile acids, enhanced FXR gene expression increases the expression of SNAI2, which in turn decreases the inhibitory effect of miR-1 on HDAC6 and HNF4α expression. Upregulated HDAC6 and HNF4α interact with each other, which further promotes upregulation of CDX2, KLF4 and MUC2, thereby promoting gastric IM development

A model for the development of BA-induced gastric IM is depicted in Fig. 6. This figure shows that BAs induce FXR upregulation in gastric cells, thereby stimulating SNAI2 activation, which leads to transcriptional inhibition of miR-1 and, finally, to transcription upregulation of downstream intestinal markers. Hence, FXR may serve as a critical regulator of the BA-induced intestinal phenotype in gastric epithelial cells. This new FXR/SNIA2/miR-1 axis may provide novel insights into the mechanism underlying the initiation and development of gastric IM. Suppression of FXR to abrogate the pathway may be a potential approach for preventing gastric IM or even GC in patients with bile regurgitation.

## Supplementary Information

ESM 1 (DOCX 452 kb)

ESM 2 (XLSX 50 kb)

ESM 3 (XLSX 20 kb)

## Declarations

### Data availability

All data generated or analyzed during this study are available.
