# Supplementary material for: Bile acids increase intestinal marker expression via the FXR/SNAI2/miR-1 axis in the stomach
Source: Cell Oncol (Dordr). 2021 Sep 12;44(5):1119–31. doi: 10.1007/s13402-021-00622-z (PMC8516775; doi:10.1007/s13402-021-00622-z)
Supplement: Supplementary file 1 — (DOCX 452 kb) [file 13402_2021_622_MOESM1_ESM.docx]

**Supplementary figures**


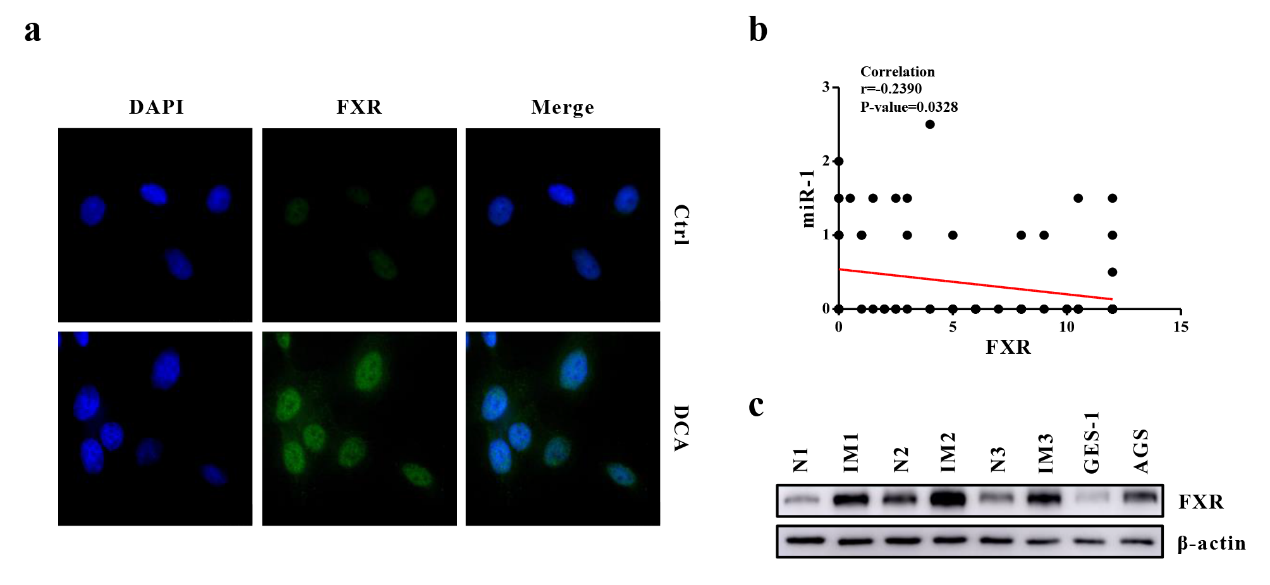


**Supplementary Information 1. a** IF staining for FXR expression in GES-1 cells treated with DCA (100 µM) for 24 hours. **b** The correlation between FXR and HDAC6 or HNF4α in IM tissue. **c** Levels of FXR was detected by immunoblot analysis in 3 paired IM and normal tissue cells, as well as GES-1 and AGS cells.


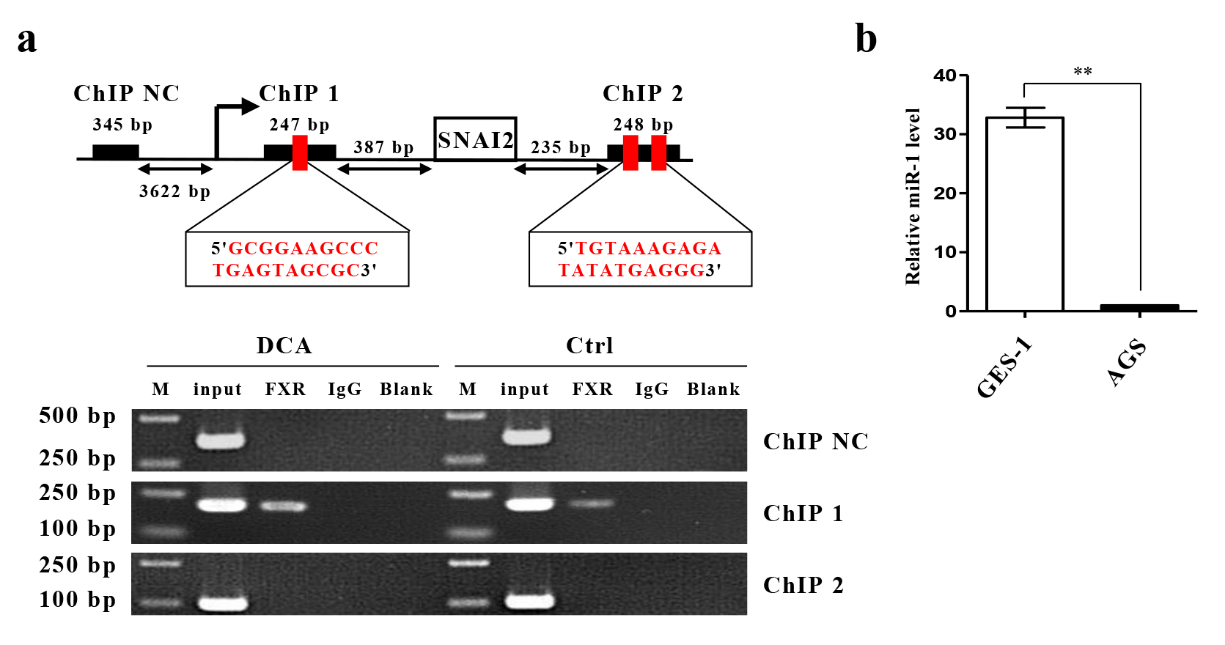


**Supplementary Information 2. a** qRT-PCR analysis showed upregulation of FXR by lentiviral vectors led to upregulation of HDAC6, HNF4α and CDX2. **b** Knockdown of FXR by small interfering RNA (siRNA) led to inhibition of downstream intestinal markers.


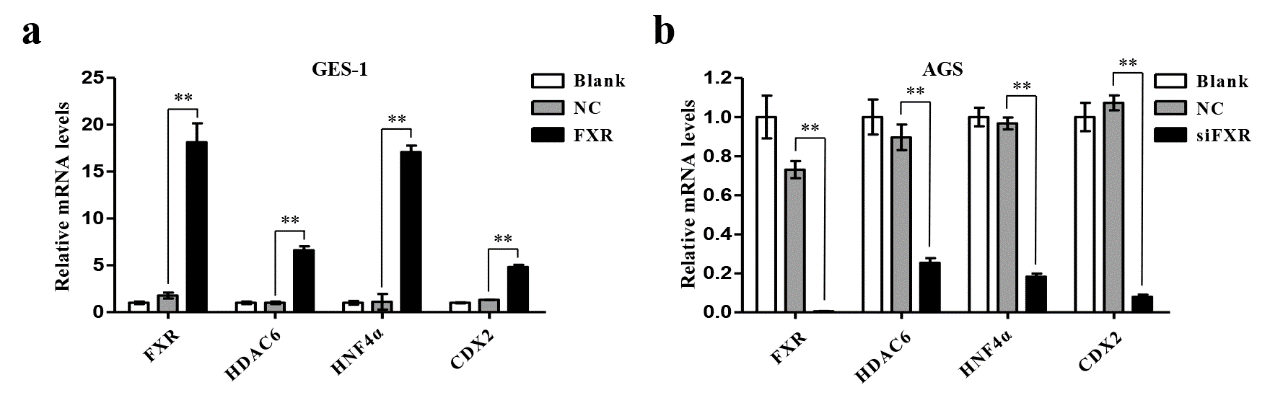


**Supplementary Information 3. a** A ChIP assay was performed to analyze the direct binding of FXR to the SNAI2 promoter in GES-1 cells treated with DCA (100 µM) for 24 hours. M: Marker. **b** qRT-PCR for miR-1 in GES-1 and AGS cells. β-actin RNA was used as internal control.
